# Immune Dysregulation in IgG_4_-Related Disease

**DOI:** 10.3389/fimmu.2021.738540

**Published:** 2021-09-01

**Authors:** Jiachen Liu, Wei Yin, Lisa S. Westerberg, Pamela Lee, Quan Gong, Yan Chen, Lingli Dong, Chaohong Liu

**Affiliations:** ^1^Department of Pathogen Biology, School of Basic Medicine, Tongji Medical College, Huazhong University of Science and Technology, Wuhan, China; ^2^Wuhan Children’s Hospital, Tongji Medical College, Huazhong University of Science and Technology, Wuhan, China; ^3^Department of Microbiology Tumor and Cell Biology, Karolinska Institutet, Stockholm, Sweden; ^4^Department of Paediatrics and Adolescent Medicine, Li Ka Shing Faculty of Medicine, The University of Hong Kong, Hong Kong, China; ^5^Department of Immunology, School of Medicine, Yangtze University, Jingzhou, China; ^6^The Second Department of Pediatrics, Affiliated Hospital of Zunyi Medical University, Zunyi, China; ^7^Department of Rheumatology and Immunology, Tongji Hospital, Tongji Medical College, Huazhong University of Science and Technology, Wuhan, China

**Keywords:** adaptive immunity, autoantigen, autoimmune disease, IgG_4_-related disease, innate immunity

## Abstract

Immunoglobin G_4_-related disease (IgG_4_-RD) is one of the newly discovered autoimmune diseases characterized by elevated serum IgG_4_ concentrations and multi-organ fibrosis. Despite considerable research and recent advances in the identification of underlying immunological processes, the etiology of this disease is still not clear. Adaptive immune cells, including different types of T and B cells, and cytokines secreted by these cells play a vital role in the pathogenesis of IgG_4_-RD. Antigen-presenting cells are stimulated by pathogens and, thus, contribute to the activation of naïve T cells and differentiation of different T cell subtypes, including helper T cells (Th1 and Th2), regulatory T cells, and T follicular helper cells. B cells are activated and transformed to plasma cells by T cell-secreted cytokines. Moreover, macrophages, and some important factors (TGF-β, etc.) promote target organ fibrosis. Understanding the role of these cells and cytokines implicated in the pathogenesis of IgG_4_-RD will aid in developing strategies for future disease treatment and drug development. Here, we review the most recent insights on IgG_4_-RD, focusing on immune dysregulation involved in the pathogenesis of this autoimmune condition.

## Introduction

Immunoglobin G_4_-related disease (IgG_4_-RD) is a group of autoimmune diseases involving fibrosis and inflammation of multiple organs and systems. This disease has three major characteristics: (i) remarkable elevated serum concentrations of IgG_4_; (ii) multiple IgG_4_^+^ plasma cells in the lesion regions; and (iii) good response to corticosteroid treatment (1, 2).

Autoimmune pancreatitis (AIP) was the first IgG_4_-RD to be described in 2001 by Japanese scientists Hamano et al. (3) In 2003, Kamisawa et al. (4) observed infiltration of IgG_4_-secreting plasma cells in extra-pancreatic organs and proposed a new clinicopathological entity that linked AIP and systemic IgG_4_-RD. Since then, an increasing number of IgG_4_-RDs have been discovered in different organs, such as the liver (5–7), kidneys (8–11), and lungs (12–14). With the deepening of research on IgG_4_-RD, an article in *Autoimmunity Reviews* (15) officially confirmed the existence of this disease in 2010. Soon afterward, the first international consensus guideline on the management and treatment was published in *Arthritis & Rheumatology* (16) in 2015. To date, the pathogenesis of IgG_4_-RD remains unclear and is thought to involve multiple factors, including adaptive and innate immunity and autoantigens. Herein, we present the most up-to-date information on immune dysregulation in IgG_4_-RD.

## Adaptive Immunity

### IgG_4_

**Figure 1:**
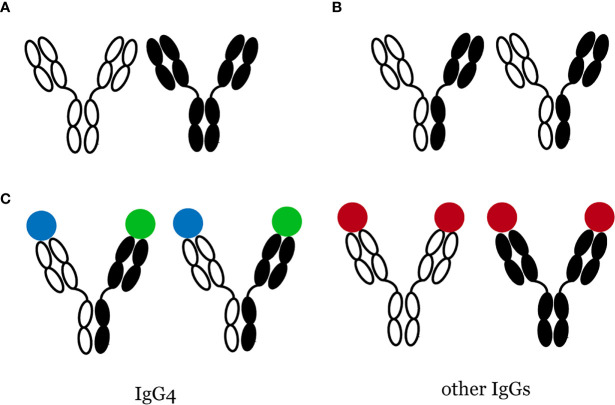
Bispecific IgG4 antibodies are produced through Fab-arm exchange. **(A)** Most IgGs do not change their structure, **(B)** Fab-arm exchange of IgG4 antibodies, **(C)** IgG4 is functional monovalency while other IgGs are not.

Among the four IgG subtypes, namely IgG_1_, IgG_2_, IgG_3_, and IgG_4_, IgG4 has the lowest concentration in normal human serum, accounting for only 5% of the total IgG levels (17). Antibodies are immunoglobin (Ig) molecules composed of two heavy (H) and two light (L) chains, both having a constant region (C_H_ or C_L_) identical for all antibodies of the same isotype and a variable region (V_H_ or V_L_) that recognizes and binds a specific antigen. Antibodies comprise two antigen-binding fragments (Fab) that bind to antigens and one constant fragment (Fc) that binds to the cell surface and allows phagocytosis. Most immunoglobin (Ig) G antibodies have these characteristics: (i) they have two identical antigen-binding sites; (ii) they do not change their structure after being secreted by plasma cells. However, Aalberse et al. (17) described IgG_4_ as an “odd antibody” because of its unique properties different from other Igs. First, it cannot cross-link identical antigens (“functional monovalency”) (18, 19). Instead, the exchange of half-molecules, also called “Fab-arm exchange” (**Figure 1**), contributes to bispecific IgG_4_ antibodies. Second, unlike other Igs, which are proinflammatory, IgG_4_ has a lower affinity for C1q (the q fragment, a part of complement C1, is the site where Igs first bind) and Fc receptor (18). These properties suggest that IgG_4_ may have anti-inflammatory activities. Remarkably increased serum IgG_4_ levels and multiple IgG_4_^+^ plasma cell infiltration are important features of IgG_4_-RD. Nevertheless, its function is still unknown in the pathogenesis of IgG_4_-RD: whether it plays a protective role in IgG_4_-RD by participating in the anti-inflammatory process, acts as a pathogenic factor mediating the occurrence of IgG_4_-RD, or is merely a manifestation induced by inflammatory stimulation, has not been determined yet. In 2015, Shiokawa et al. (20) reported an interesting finding that pancreatic injury could be induced in neonatal male Balb/c mice by injecting patient IgG_1_ or IgG_4_, and the injury caused by IgG_1_ was more serious. However, the pathogenic activity of IgG_1_ and the severity of pancreatic injury were substantially inhibited by simultaneously injecting IgG_4_. Moreover, rituximab, a monoclonal antibody, specifically binds to cluster of differentiation 20 (CD20), a biomarker of pre-B and mature B cells, and exerts cytotoxic, anti-proliferative, and apoptotic effects. A prospective, open-label trial by Carruthers et al. (21) discovered that rituximab could relieve the symptoms of IgG_4_-RD. However, after rituximab treatment for 12 months, 19 patients with elevated baseline IgG_4_ levels showed a marked decrease in IgG_4_, but only 42% achieved normal IgG_4_ levels. This finding showed that rituximab might not work by decreasing serum IgG_4_ levels but by depleting activated B cells; thus, IgG_4_ might not have a considerable role in IgG_4_-RD. Similarly, Gauiran et al. (22) examined two cases of IgG4 myeloma, of which both showed high serum IgG4 levels, but neither of them manifested typical IgG_4_-RD presentations.

### B-Lymphocytes

IgG_4_-RD is characterized by remarkably elevated serum IgG_4_ levels and expansion of lymphoid follicles. However, a wide range of IgG_4_ concentrations have been observed in patients with IgG_4_-RD, with some reaching levels 30 times over the upper limit, but up to 40% of patients show normal IgG_4_ levels (23). Next-generation sequencing examinations have led to the identification of a large number of circulating, antigen-specific plasmablasts in the peripheral blood of patients with IgG_4_-RD (24). Flow cytometry studies have confirmed a substantial increase in the number of plasmablasts in the blood of IgG_4_-RD patients (24). Over 95% of B-lymphocytes (except plasmablasts and plasma cells) express CD20, which makes rituximab, an anti-CD20 drug, effective in eliminating B cells from patients with IgG_4_-RD (25). CD19 has a broader expression than CD20 and is found in all B cell lines except plasma cells (26). B cell depletion leads to a decrease in serum IgG_4_ concentrations, indicating the preponderance of short-lived plasmablasts and plasma cells in regulating serum IgG_4_ levels. However, the depletion of B cells does not cause a complete normalization of IgG_4_ concentration, which suggests that long-lived plasma cells might maintain the production of antibodies. Mattoo et al. (24) examined 84 patients diagnosed with IgG_4_-RD and found that the number of plasmablasts was higher than the control group even in patients with normal serum IgG_4_ concentration. Besides, patients who undergo a relapse after rituximab treatment exhibit an increase in the number of plasmablasts and stronger somatic hypermutation. Therefore, peripheral plasmablasts might be considered as a biomarker for IgG_4_-RD (27).

B-lymphocytes are also involved in the fibrosis of diseased tissues. Della-Torre et al. (28) cultured naïve B cells, CD19^+^ B-lymphocytes, memory B cells, or plasmablasts from IgG_4_-RD with human fibroblasts and observed that the B cells and plasma cells from IgG_4_-RD patients can promote collagen synthesis in fibroblasts by secreting the pro-fibrotic molecules of platelet-derived growth factor B. They also observed those cells could induce the remodeling of the extracellular matrix (ECM) by producing ECM-crosslinking enzymes, such as lysyl oxidase-like 2, and chemotactic factors CCL-4, CCL-5, and CCL-11. This finding suggests that B cells might be directly involved in tissue fibrosis in IgG_4_-RD.

### T Lymphocytes

T cells are formed in the bone marrow but mature in the thymus. They are involved in cell-mediated immunity and have three major types: helper T cells (Th), cytotoxic T cells (Tc), and regulatory T cells (Treg). Th cells can be subdivided into mutually exclusive Th1 and Th2 subsets. The Th1/Th2 balance has been confirmed to participate in autoimmune and allergic diseases (29, 30).

#### Th2 Cells

Th2 cells primarily secrete interleukin-4 (IL-4), IL-5, and IL-13 that participate in a plenty of pathogenic phenomena, such as allergic reaction, hypersensitivity, and IgE and IgG class switching (31). Because extremely high levels of IgG_4_ are observed in nearly all patients with IgG_4_-RD, it is one of the key diagnostic criteria for this disease. In 2005, Schmitz et al. (32) reported that IL-33 activates NF-κB and MAP kinases *via* IL-1 receptor ST-2 and thus promotes the production of cytokines from Th2 cells. In 2007, Miyake et al. (33) observed a disrupted balance between Th1/Th2 cells and increased peripheral Th2 levels in a patient with Mikulicz syndrome. In 2010, Akitake (34) and Suzuki (35) discovered that the number of Th2 cells and the levels of cytokines produced by them (IL-4, IL-5, and IL-13) were higher than normal in diseased tissues. In 2012, Tanaka et al. (36) examined 15 patients with Mikulicz disease and discovered that Th2-mediated adaptive immunity was essential in IgG_4_-RD. In 2013, GATA3^+^ Th2 cells are proved to be present in IgG4-related sclerosing cholangitis and type 1 autoimmune pancreatitis. Zen et al. (37) found the ratio of GATA3^+^/T-bet^+^ cell is shifted towards Th2, which may result in the recruitment of lymphocytes in patients with IgG4-RD.

However, a recent study questioned this conclusion. Maehara et al. (38) found that IL-4 mRNA levels were markedly high, whereas CD4^+^GATA3^+^ Th2 cells and GATA3 mRNA levels were low in patients with IgG_4_-RD, and that CD4^+^GATA3^+^ Th2 cells primarily exist in patients with allergic reaction. They explained that increased IL-4 levels might be produced by non-Th2 cells. Another puzzling finding, as reported by Okazaki et al. (39), was that instead of IL-4 levels, interferon (IFN)-γ levels were increased, and Th1-mediated immune response seems involved in IgG_4_-RD AIP. Another study, however, examined 44 patients with chronic periaortitis, one of the subtypes of IgG4-RD, and demonstrated the elevated level of CXCL12 and dominant infiltration of GATA3^+^ Th2 cells. This novel finding indicates CXCL12 might drive fibrocytes accumulation and Th2 differentiation (40).

#### Th1 Cells

In 1986, the two types of helper T cells, Th1 and Th2, were first described by Mosmann (41). Since then, various Th subsets with different functions have been discovered and reported. Th1 cells express CD4 and are activated by IL-12 and IFN-γ.

Recently, some researchers found elevated Th1 levels in IgG_4_-RD and speculated that Th1 cells might participate in the progression of IgG_4_-RD. Ohta et al. (42) observed a considerable increase in the population of Th1, but not Th2 cells in patients with IgG_4_-related sclerosing sialadenitis. Besides, the peripheral serum level of IFN-γ was substantially elevated.

#### CD4^+^ CTLs and CD8^+^ CTLs

However, multi-color immunofluorescence proved that high IFN-γ levels were derived from novel CD4^+^ cytotoxic T cell (43). Granzyme B (GZMB), one of the effector molecules of CD4^+^ T cells, was also present in the peripheral blood, whereas CD4^+^GZMA^−^IFN-γ^+^ Th1 cells were rare in diseased tissue. CD4^+^ CTLs are widely distributed in humans (44, 45) and mice (46, 47). They recognize antigenic peptides and target cells through MHC class II (MHC-II)- and HLA-II-dependent antigen-specific pathways, respectively (45), and perform their killing functions by secreting GZMB and perforin. Their accumulation has also been seen in some autoimmune diseases, and their severity positively correlated with the number of CD4^+^ CTLs (48). Mattoo et al. (43) reported that proinflammatory factors, such as transforming growth factor (TGF)-β, IL-1β, and IFN-γ, were increased in peripheral blood, suggesting the potential role of CD4^+^ CTLs in tissue fibrosis. Recently, a hypothesis in the mechanism of fibrosis and inflammation in IgG4-RD suggests that self-reactive cytotoxic CD4 T cells might be activated by T cells and induce cell programmed death, thus causing tissue fibrosis and inflammation (49).

Perugino et al. (50) observed CD27loCD28loCD57hi cells are dominant effector subset among circulating CD4^+^ CTLs in IgG4-RD, showing significant clonal expansion and different gene expression. Besides, they found marked infiltration of granzyme A-expressing CD8^+^ CTLs in diseased tissue and expansion of effector/memory CD8^+^ T cells in blood samples. Tissue studies also proved that apoptosis was common in diseased tissue, with a high proportion of nonimmune, nonendothelial mesenchymal cells.

In IgG4-related disease, presumably self-reactive cytotoxic CD4 T cells infiltrate tissues, are reactivated by T cells and induce apoptotic death. Molecules secreted by activated B cells and by CD4^+^ CTLs drive an exaggerated wound healing response resulting in fibrosis and compromised tissue function.

#### Treg Cells

Regulatory T cells (Treg) are CD4^+^CD25^+^ T cells that primarily secrete the anti-inflammatory factor IL-10 (51) and the fibrogenic factor TGF-β to maintain immune tolerance and immune homeostasis *in vivo*. Treg cells can be divided into two broad subsets: thymus-derived CD4^+^CD25^+^ forkhead box protein 3 (FOXP3)^+^ natural Treg cells and periphery-generated induced Treg (iTreg) cells. There are three main subtypes of iTreg cells: (i) CD4^+^FOXP3^+^ iTreg cells, (ii) CD4^+^FOXP3^-^ IL-10-producing type I Treg (Tr1) cells, and (iii) TGF-β-expressing T_H_3 cells.

In 2018, Lin et al. (52) observed that IL-10 does not affect IL-4-induced IgE production but causes a 20-fold increase in IgG_4_ production in B cell cultures, whereas the production of both IgE and IgG_4_ was promoted by IL-10 in peripheral blood mononuclear cell (PBMC) cultures. Furthermore, they observed that IL-10 could diminish IL-4-induced IgE production without affecting the production of IgG_4_. Similarly, Punnonen et al. (53) proved that IL-10 decreases IgE production by IL-4-stimulated PBMCs. Another study reported that the blockage of IL-10 receptors in CD4^+^CD25^+^ Treg cells caused a decrease in their IgE-suppressing and IgG_4_-inducing effects (31).

TGF-β, a regulatory cytokine, is involved in the suppression of immune reactions. It can induce fibroblast transformation into myoblasts, increase type I collagen synthesis, and inhibit collagenase synthesis by Smad signaling pathways, thus promoting tissue fibrosis (54–56).

It is believed that Treg cells play an important role in the pathogenesis of IgG_4_-RD (57). Plenty of histological examinations have shown that an increased number of Treg cells are associated with different IgG_4_-RDs, including, but not limited to, AIP (58), IgG_4_-related sclerosing cholangitis (59), and Mikulicz’s syndrome (36). Therefore, it can be believed that most target organs in IgG_4_-RD have Treg cell infiltration. Besides, the level of Treg cells is elevated not only in the diseased tissue but also in peripheral blood (60). Miyoshi et al. (61) analyzed circulating Tregs in AIP and found CD4^+^CD25^+^ Tregs markedly elevated in AIP patients while naïve Tregs decreased, which indicates changes of Tregs might affect IgG4 production and disease progression.

Further studies showed that the levels of IL-4, IL-10, and Foxp3 were positively correlated with IgG_4_/IgG, suggesting that Treg-mediated immune response could promote IgG_4_ production and IgG_4_-RD progression.

#### Th17 Cells

Upon activation with TGF-β, IL-6, and IL-23, CD4^+^ Th cells can differentiate into Th17 cells. Some researchers discovered that IL-17, an inflammatory cytokine, has a strong effect on resting stromal cells and might be involved in fibrosis (62–64). Feng et al. (65) reported that IL-17 could promote the synthesis and secretion of collagen through the TGF-β signaling pathway and regulate the infiltration of fibroblasts. Ohta et al. (42) observed a correlation between the expression of IL-17 and elevated number of Th1 and Tc1 cells in IgG_4_-related sclerosing sialadenitis. They subsequently proposed a hypothesis that IL-17, with Th1 and Tc1 cells, could cause elevated serum levels of IgG_4_ and IL-17 and numbers of Th1 and Tc1 cells, but not Th2 and Tc2 cells. However, the exact role of IL-17 in the pathogenesis of IgG_4_-RD is still a mystery.

#### T Follicular Helper (Tfh) Cells

Tfh cells are specialized CD4^+^ T cells involved in the formation of a germinal center (GC), where B cells development and selection of antibodies occur (66). A GC contains a dark zone where B cells proliferate, and a light zone, where Tfh and B cells interact (67). A large number of ectopic GCs can be found in the pathological tissues of IgG_4_-RD. A study reported that nearly 70% of CD4^+^ T cells in the lesion of IgG4-related salivary gland are Tfh cells (68), and they play an important role in driving plasma cell and plasmablast differentiation *via* Tfh cytokine IL-21 (69). Besides, IL-4 has been proved to be involved in IgG4 class-switching in IgG4-RD both *in vitro* (70) and *in vivo* (71). A functional analysis suggested that IL-4-secreting Tfh cells assisted in antibody class-switch (72), whereas IL-21-secreting Tfh cells were essential for somatic hypermutation of B cells (73). Moreover, GCs exist even without Th2-related genes (74) but disappear without both IL-4 and IL-21 receptors (69). Zaidan et al. (75) observed Tfh cell infiltration of the GC light zone, which was unique to IgG_4_-RD. In 2011, Maehara et al. (76) examined the ectopic formation of GC and the expression of IL-21, Th2-, Th17-, and Tfh-related cytokines in 12 patients with Mikulicz’s syndrome and found that IL-4-expressing Tfh cells were primarily located outside of the ectopic GCs, whereas IL-21-expressing Tfh cells were located inside. Similarly, the upregulated expression of IL-21 mRNA was associated with the formation of ectopic GCs (76, 77). Thus, GC formation, B cell selection, and IgG_4_ antibody class-switch *via* different Tfh cell-produced cytokines are basic pathological events in the progression of IgG_4_-RD.

Based on the different expression levels of chemokine receptors CXCR3 (chemokine (C-X-C motif) receptor 3) and CCR6 (chemokine (C-C motif) receptor 6), Tfh cells can be divided into three subsets: Tfh1 (CXCR3^+^ CCR6^-^), Tfh2 (CXCR3^-^ CCR6^-^), and Tfh17 (CXCR3^-^ CCR6^+^) (78). Recently, several studies confirmed the expansion of circulating plasmablasts (24, 27, 79, 80) and the Tfh2 cells (81, 82) in IgG_4_-RD. Moreover, the number of Tfh2 cells positively correlated with the serum IgG_4_ concentration (81, 82) and proportion of IgG_4_^+^ plasma cells in diseased tissues (83). In vitro studies have shown that Tfh2 cells promote the differentiation of plasmablasts (81). Interestingly, Akiyama et al. (81) observed that the glucocorticoid therapy decreased the number of activated Tfh2 cells, which increased again during disease relapse, suggesting that activated Tfh2 cells might be used as a biomarker for IgG_4_-RD.

Another finding showed Tfh1 cells were activated in IgG_4_-RD but did not affect the production of IgG_4_ antibody (81). Therefore, the effect of Tfh1 cells remains to be further elucidated.

T cell immunoreceptor with immunoglobulin and ITIM domain (TIGIT), a co-inhibitory receptor discovered recently (84), was thought to be a novel Tfh marker. Akiyama et al. (85) analyzed the expression of TIGIT in peripheral CD4^+^T cell subsets and found that peripheral Tfh cells have higher expression of TIGIT than Th cells. They also observed that TIGIT^+^ Tfh cells secretes more IL-21 than TIGIT^-^ Tfh cells, which could be used to trace the progression of IgG4-RD.

Tfh 17 cells do not appear to be involved in IgG_4_-RD because their numbers do not vary with serum IgG_4_ levels (81). Moreover, IL-17, a marker of Tfh17 cells, was rarely expressed in diseased tissues (76).

## Innate Immunity

In the past few years, innate immunity in IgG_4_-RD has gradually attracted researchers’ attention. Toll-like receptor (TLR), a key receptor that belongs to the pattern recognition receptor family, can bind to pathogen-associated molecular patterns (PAMPs), and activate inflammatory factors through the NF-κB and MAPK pathways. Other receptors, such as nucleotide-binding oligomerization domain (NOD)-like receptor (NLR) and C-type lectin receptor, can identify PAMPs and induce immune reactions.

### Macrophages

Macrophages are relatively long-lived phagocytic cells of mammalian tissues derived from blood monocytes. Based on their phenotype and function, activated macrophages can be divided into two main categories, classically activated M1 macrophages and alternatively activated M2 macrophages, which are further divided into pro-allergic M2a, immune-regulatory M2b, and M2c types (86, 87).

When cultured with GM-CSF, monocytes can differentiate into M1 macrophages (88). Some important factors, including bacterial lipopolysaccharide (LPS) (89), monosodium urate monohydrate (90), inflammatory biomarker C-reactive protein (CRP) (91), and Th1 cytokines IFN-γ and tumor necrosis factor-α (TNF-α), promote the production of M1 macrophages. Activated M1 macrophages secrete proinflammatory factors and mediate adaptive immunity, thus eliminating the pathogens that damage normal host tissues. Hong et al. (92) observed markedly increased levels of TNF-α in submandibular glands of patients with IgG_4_-related sialadenitis (IgG_4_-RS). Besides, TNF-α treatment showed a consistent redistribution of the transcription factor EB in patients with IgG_4_-RS. This finding suggests that TNF-α suppresses autophagic flux and lysosomal dysfunction and causes injury of acinar cells through the ERK1/2 pathway. To sum up, M1 macrophages might participate in the formation and progression of IgG_4_-RS.

Interestingly, the anti-inflammatory M2 macrophages also participate in the IgG_4_-mediated immune response. Usually, M2 macrophages become polarized by the stimulation of Th2-derived IL-4 and IL-13 (93). IL-33, a member of the IL-1 family, can amplify IL-13-induced M2 macrophage polarization (94). However, inflammatory monocytes can differentiate into M2 macrophages *via* basophil-derived IL-4 during an allergic reaction (95). Watanabe et al. (96) showed that the TLR signaling pathway could enhance immune dysregulation in IgG_4_-RD. Besides, Ishiguro et al. (97) observed that TLR-7-, TLR-8-, and TLR-9-related genes were overexpressed in IgG_4_-RD. Chang et al. (98) reported that *in vitro* stimulation with TLR-7 agonist could increase IL-33 production by alveolar macrophages in virus-infected lung tissues. Baenziger et al. (99) proved that, besides being involved in antiviral infection, TLR-7 is involved in acquired immunity. Mice experiments confirmed that the activation of TLR-7-expressing plasmacytoid dendritic cells could lead to arthritis and lupus nephritis (100). These interesting findings suggest that virus infection and/or endogenous RNAs could initiate the formation of IgG_4_-RD. Pathogens activate IL-33-secreting M2 macrophages *via* TLR-7 and thus promote the production of Th2 cytokines, leading to tissue fibrosis and IgG_4_ class switching. Bianchini et al. (101) further examined the phenotype of M2 macrophages and found that pro-allergic M2a macrophages could be converted into immune-regulatory M2b macrophages by IgG_4_ to maintain a tolerogenic state.

Another crucial macrophage involved in IgG4-RD is CCL-18-producing M2 macrophages. CCL-18, produced from activated M2 macrophages, plays an critical rule in the formation of collagen (102). DNA microarray analysis also proved that CCL-18 was upregulated in IgG4-RD (103). Furukawa et al. (104) examined 7 patients with IgG4-related dacryoadenitis and sialoadenitis and found the level of CD163, one of the markers of M2 macrophage, was significantly higher than that in Sjögren’s syndrome and healthy subjects. Similarly, Takanashi et al. (105) observed massive infiltration of CD163^+^ M2 macrophages in the diseased tissue. CD163 is also co-localized with IL-10 and CCL-18 in the fibrotic region, which indicates CCL-18-secreting M2 macrophages might be involved in the development of fibrosis in IgG4-RD. Thus, CCL-18 might be a useful biomarker for tracing the severity of IgG4-RD (106).

Human IgG antibodies bind to different members of the Fcγ receptors (FcγRs) family, which have a low affinity for IgG and thus bind merely to immune complexes (IC) (107). It is widely believed that the interaction between IgG and FcγRs stimulates the immune system by triggering the phosphorylation of immunoreceptor tyrosine-based activation motif (ITAM) of FcγR (108) and inhibition of signaling pathway by coupling with FcγR IIB (109) and IgG_4_ is commonly thought to be not compatible with FcγR (57, 110). However, Bianchini et al. (101) reported that IgG_4_ might bind to FcγRIIb on M2a macrophages and cause M2b subtype conversion, leading to IL-10 and CCL1 secretion. IL-10 further contributes to class switching of IgG_4_-secreting B cells, whereas CCL1 recruits CCR8^+^Foxp3^+^ Tregs from the periphery. Growing evidence indicates that ITAM-containing FcγR also has an inhibitory intrinsic ability (111). Boekhoudt et al. (112) reported that IFN-γ signaling pathways are inhibited by IC-mediated signaling through FcγR I and M2-like macrophages can be induced by IgG_4_ through FcγR I (101).

The fate of organs in inflammation and injury is controlled by the balance of M1/M2 macrophages. When the infection or inflammation caused by those pathogens is severe enough to affect the target organ, macrophages are activated and differentiated to the M1 subtype to antagonize the stimulation by Th1 cytokines. However, if M1 macrophage-mediated immune responses continue, it could cause pathological damage to the tissue. Therefore, M2 macrophages express IL-10 and TGF-β and trigger Th2-mediated reaction to suppress the immune response and promote damaged tissue repair.

### Basophils and Eosinophils

During parasitic infections, allergic reactions, and autoimmune diseases, basophil cells are recruited into tissues where they produce Th2 cytokines and participate in the immune response of Th2 cells as antigen-presenting cells (APCs) (113). APCs and T cells secrete Th2 cytokines IL-4, IL-10, and IL-13 to induce IgG_4_ production. Several studies show that a combination of basophils and microbial antigens induces the production of Th2 cytokines (114, 115). Therefore, exogenous stimulation may facilitate the occurrence of IgG_4_-RD through the activation of TLRs in basophils. Watanabe et al. (96) proved that microbial antigens activate TLRs and NLRs in monocytes to induce IgG_4_ secretion by activating of B cell-activated factor (BAFF)-mediated pathways. They also examined IgG_4_ and cytokine responses to various NLR and TLR ligands and found that the activation of TLRs in basophils promotes the secretion of IgG_4_, BAFF, and IL-13 and thus leads to the progression of IgG_4_-RD (116). This finding suggests that TLR-mediated basophil activation could facilitate disease development through the BAFF signaling pathway.

Eosinophils are involved in the pathogenesis of inflammation (117). They secrete various cytokines and affect T cell expansion and Th1/Th2 cell polarization (118). Some reports have shown a connection between eosinophils and IgG_4_-RD (119–121). Some researchers also observed that patients with type I AIP, a typical presentation of IgG_4_-RD, have a long history of allergies (122, 123). Peripheral eosinophils and serum IgE were elevated in these patients. However, when they tried to determine the allergen by skin prick test or specific IgE quantitation, the allergen sensitization profile failed to reveal the culprit. Furthermore, they tested the mean IgE levels and eosinophil counts and found no connection between those data and the atopic state of patients. This finding suggests that elevated peripheral eosinophil counts and IgE levels are the intrinsic characteristics of IgG_4_-RD. TGF-β, one of the major cytokines secreted by eosinophils (118), participates in the formation of tissue fibrosis. Moriyama et al. (123) observed a positive correlation between peripheral eosinophil counts and treatment-free disease duration in IgG_4_-RD, which indicated that eosinophils might promote the end-stage development of the disease and participate in the pathogenesis of fibrosis.

Eotaxin-3, or CCL-26, is recently considered to be a potent chemoattractant for eosinophils (124). To date, Eotaxin-3 is thought to have eosinotactic activity both *in vitro* and *in vivo*. IL-4 and IL-13 are potent co-inducers of Eotaxins in epithelial and endothelial cells, consistent with Th2 responses in allergic and eosinophilic diseases (125). Eotaxin-3 could also attract eosinophils, basophils, and killer T cells *via* receptor CCR3 and CX3CR1. Consequently, increased local expression of Eotaxins has been described in various eosinophilic diseases. In 2021, Takanashi et al. (126) analyzed proteins overexpressed in patients with IgG4-RD with lymphadenopathy and discovered that this disease was linked with eosinophilia and Eotaxin-3 could be thought to be a potent biomarker.

Lymphadenopathy in IgG4-RD represents a phenotype associated with high disease activities, eosinophilia and relapsing disease. Eotaxin-3 is a novel biomarker related to IgG4-RD with lymphadenopathy.

### Complement Activation System

The complement system participates in developing IgG_4_-RD. It contains a cascade of proteins that lead to the lysis of microorganism-infected cells. The complement system can be activated by three pathways: the classic, alternative, and lectin pathways. These three pathways converge on the production of C3 convertase, an enzyme that triggers the cleavage of C3 into an enzymatically active C3b and an anaphylatoxin C3a that could mediate inflammatory responses. Saeki et al. (127) examined 10 patients with hypocomplementemia of unknown etiology and found six to have high serum IgG_4_ levels. Kawano et al. (128) identified 22 patients (53.7%) with hypocomplementemia among 41 IgG_4_-related kidney disease (IgG_4_-RKD) patients. Besides, 16 showed lower levels of C3, C4, and CH50. IgG_4_-RKD is now considered as a complement-triggered inflammatory disease (129). A native renal biopsy showed that ethnic factors might contribute to different incidence rates of IgG_4_-RKD (**Table 1**). Besides, renal interstitial tissue fibrosis is now recognized as an inevitable process of end-stage renal disease. Wang et al. (135) examined serum C3 and C4 levels and found that these levels were negatively correlated with the number of infiltrated IgG_4_-secreting plasma cells in the kidney. However, the role of IgG_4_ in complement activation remains unclear. In 2006, Muraki et al. (136) found that IgG1 levels were considerably increased, whereas C3 and C4 levels were reduced in AIP, which indicated that IgG1, instead of IgG_4_, was involved in the activation of the classic pathway. In 2007, Kolfschoten et al. (18) speculated that IgG_4_ might be a protective (anti-inflammatory) antibody because of its unique “Fab-arm exchange” property. In contrast, in 2016, Sugimoto et al. (137) observed that in IgG_4_-RD patients with hypocomplementemia, IgG_4_ might participate in the activation of the complement system.

**Table 1:** Five studies from America, South Korea, Japan, Australia, and India show the involvement of a potential ethnic factor in the morbidity of IgG_4_-RD.

Country	Kidney biopsy	IgG_4_-RKD	IgG_4_-RKD/kidney biopsy (%)
America (130)	4492	2	0.04
South Korea (131)	5174	12	0.23
Japan (132)	6978	47	0.67
Australia (133)	1238	12	0.97
India (134)	4000	11	0.28

Further exploration on the function of the complement system in IgG_4_-RD is necessary.

## Autoantigens

Antigens that induce an immune response in patients with IgG_4_-RD have not been identified to date. However, four potential autoantigens have been discovered: prohibitin, annexin A11, laminin 511-E8, and galectin-3.

In 2015, Du et al. (138) examined the sera of 89 patients with IgG_4_-RD and found that 73% were reactive with prohibitin, whereas only 1.4% of the healthy control group were positive. Prohibitin participates in the progression of many diseases (139, 140) and may function as a tumor suppressor and promote anti-proliferative activity by inhibiting cell cycle and DNA synthesis. ELISA showed that the levels of anti-prohibitin in the sera of IgG_4_-RD patients were substantially higher than those in the control group, suggesting that anti-prohibitin antibodies might contribute to the enlargement of diseased organs in IgG_4_-RD. Zhou et al. (141) found that the expression of prohibitin was markedly lower in the renal tissue of rats with unilateral ureteral obstruction and renal tubule interstitial fibrosis. This finding suggests that prohibitin may be involved in tissue fibrosis. Another study showed low levels of prohibitin in inflammatory bowel disease and may be conducive to reduce pain (142). Subsequently, Hubers et al. (143) found a novel autoantigen annexin A11 in IgG_4_-RD and thought IgG_4_ might perform its anti-inflammatory function by blocking the binding of IgG1 to annexin A11. Annexin A11 is a calcium-dependent phospholipid-binding protein abundant in the nucleus. When cell damage occurs, exposed annexins are recognized as autoantigens, resulting in autoimmune diseases (144, 145). Shiokawa et al. (146) also found another auto-antibody anti-laminin 511-E8 in the serum of patients with IgG_4_-related pancreatitis. An ELISA showed this antibody was present in 26 of 51 AIP patients, but only in two of 122 healthy controls. Besides, mice immunization by injecting laminin 511-E8 can induce symptoms like IgG_4_-associated pancreatitis. However, Liu et al. (147) reported that anti-laminin 511-E8 was present in only 7% of Caucasian patients with IgG_4_-RD, which indicated that ethnic factors might play an important role in the formation of autoantigens. In 2019, Perugino et al. (148) examined the Ig gene sequence from single-cell clones in IgG_4_-RD using mass spectrometry and identified galectin-3 as an antigen recognized by IgG_4_ and IgE. The anti-galectin-3 autoantibodies were primarily IgG_4_ (28%) and IgE (11%) isotypes. Galectin-3 is expressed in various cells, including macrophages, tumor cells, eosinophils, and myofibroblasts, among which activated macrophages are the primary source. Galectin-3 has a variety of biological functions. Besides promoting cell proliferation, inhibiting apoptosis, mediating cell adhesion, and participating in the inflammatory response, it is involved in the fibrosis of the liver, kidney, lung, and other organs (149). Salah et al. (150) reported increased serum levels of galectin-3 in IgG_4_-associated pancreatitis, suggesting that galectin-3 might participate in target organ fibrosis.

However, in 2020, a large, clinically diverse cohort study of patients with IgG_4_-RD presented an interesting finding. The antibody response frequency for the autoantigens prohibitin, annexin A11, laminin 511-E8, and galectin-3 were 10%, 12%, 7% and 28%, respectively (147). Further studies are needed to identify the dominant autoantigen in IgG_4_-RD.

A recent study investigated another autoantibody, anti-IL-1 receptor antagonist (IL-1RA), by sequencing plasmablast antibody repertoires. Compared with the control group, patients with IgG4-RD showed an increased level of plasma responses to IL-1RA, which neutralized the activity of IL-1RA and thus caused inflammation and fibrosis. This finding indicated a novel immunologic mechanism in IgG4-RD (151).

## Microbial Species

Gut microbes are essential for the development and activity of the immune system. Microbial antigens are recognized by the CD4^+^ T cells *via* MHC II molecules. Gut bacterial strains stimulate the expansion of a variety of immune cell populations and provide signaling molecules for anti- and pro-inflammatory responses locally and systemically. Therefore, disorders of gut microbes are linked to a variety of diseases.

In 2009, an Italian research observed 90% of IgG4-related pancreatitis could detect peptide AIP(1-7), which shows homology with plasminogen-binding protein (PBP) of *Helicobacter pylori* and with ubiquitin-protein ligase E3 component n-recognin 2 (UBR2) of acinar cells in the pancreas. Besides, anti-PBP antibodies were detected in 95% of AIP patients (152). So, *Helicobacter pylori* infection may induce IgG4-RD through molecular mimicry or antibody cross-reaction. However, a prospective UK cohort questioned this finding: they tested 69 patients with IgG4-RD and found there was no difference in the exposure to *H. pylori*, cytokine response, and immunological memory to PBP. Therefore, whether *Helicobacter pylori* is involved in the formation of IgG4-RD remains to be studied (153).

In 2021, A recent unique microbial species using metagenomics shows reduction of normal flora and expansion of potential pathogenic bacteria: some species such as *Alistipes*, *Bacteroides*, and butyrate-producing bacterial were depleted while pathogenic *Clostridium* and typical oral *Streptococcus* were significantly overabundant. Another interesting finding is that the level of *Eggerthella lenta*, a Th17-activating strains, was increased in IgG4-RD. This finding indicates that rare autoimmune diseases, such as IgG4-RD, could be induced by microbiome-driven immune cell types differentiation (154).

## Conclusions

**Figure 2:**
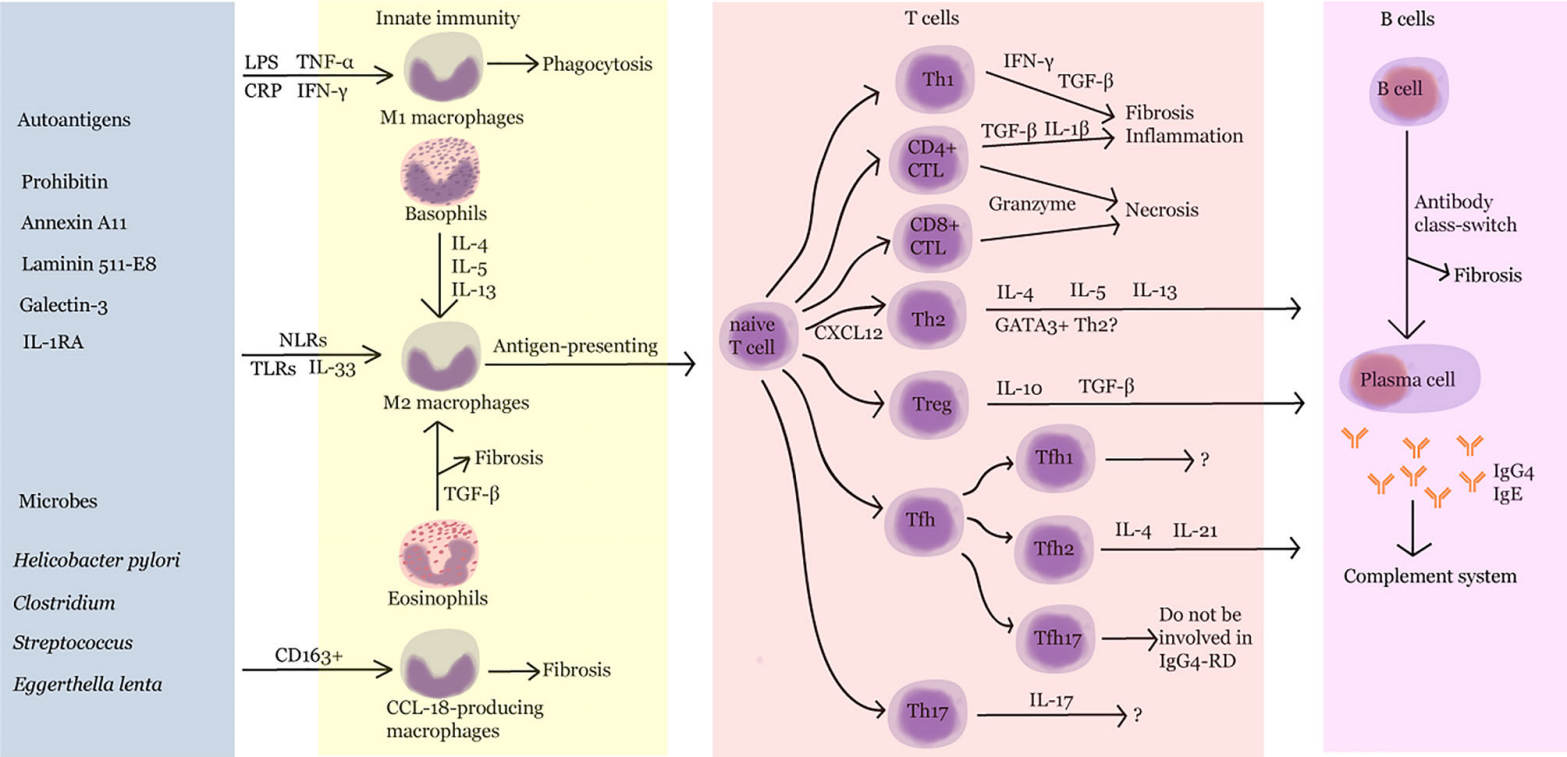
Pathogenesis of IgG4-RD. LPS, lipopolysaccharide; TNF-α, tumor necrosis factor-α; CRP, C-reactive protein; IFN-γ, interferon-γ; IL, interleukin; NLR, nucleotide-binding oligomerization domain (NOD)-like receptor; TLR, Toll-like receptor; TGF-β, transforming growth factor-β; Th, T helper; CTL, cytotoxic T lymphocyte; Treg, regulatory T cell; Tfh, follicular helper T cell.

With AIP as the first case of IgG_4_-RD, as reported by Sarles et al. in 1961, the understanding of the diagnosis and treatment of IgG_4_-RD has gradually deepened. This article reviews the studies on synergic regulation between B and T cells, cross-interaction of innate and adaptive immunity, IgG_4_ class switching, and the role of complement system in the progression of IgG4-RD (**Figure 2**). However, the knowledge regarding its pathogenesis is still limited. Further exploration of the mechanism of IgG_4_-RD will help find potential therapeutic targets and provide innovative ideas for the diagnosis and treatment of and drug development in IgG_4_-RD.

## Author Contributions

J.L. wrote the article and drew the figures. W.Y., L.W. and P.L. reviewed the paper. Q.G., Y.C., L.D., and C.L. organized and revised the paper. All authors contributed to the article and approved the submitted version.

## Funding

This work was supported by HUST Academic Frontier Youth Team (2018QYTD10) and Independent Innovation Research Fund of Huazhong University of Science and Technology (2020kfyXGYJ017).

## Conflict of Interest

The authors declare that the research was conducted in the absence of any commercial or financial relationships that could be construed as a potential conflict of interest.

## Publisher’s Note

All claims expressed in this article are solely those of the authors and do not necessarily represent those of their affiliated organizations, or those of the publisher, the editors and the reviewers. Any product that may be evaluated in this article, or claim that may be made by its manufacturer, is not guaranteed or endorsed by the publisher.
